# Early sleep intervention for improving infant sleep quality: a randomized controlled trial, preliminary result

**DOI:** 10.1186/s12887-024-04771-6

**Published:** 2024-05-04

**Authors:** Auraya Sinthong, Dussadee Ngernlangtawee

**Affiliations:** Department of Pediatric, Faculty of Medicine Vajira Hospital, Navamindradhiraj University, Bangkok, 10300 Thailand

**Keywords:** Infant sleep, Sleep intervention, Sleep quality, Night waking

## Abstract

**Background:**

Healthy sleep issues should provide to family within first 6 months of infant’s life. This study aimed to evaluate the effect of early sleep intervention on nighttime sleep quality.

**Methods:**

Eligible infants aged 4 months ± 2 weeks were randomized to receive early sleep intervention or usual care. Data on sleep variables were obtained via parental interview at baseline and 6 months of age. Using logistic regression to analyze the efficacy of early sleep intervention.

**Results:**

At baseline, 335 eligible infants were enrolled and randomized. In total, 306 participants were final analyzed: early sleep intervention group (*n* = 148) and the usual care group (*n* = 158). The early sleep intervention group had a significantly longer nighttime sleep duration and a shorter night waking duration than the usual care group (585.20 ± 80.38 min vs. 496.14 ± 87.78 min, *p* < .001 and 61.01 ± 36.38 min vs. 89.72 ± 45.54 min, *p* < .001). At 6 months of age, the early sleep intervention group had a longer night sleep duration (≥ 4 h/time) than the usual care group (adjusted odds ratio: 2.39, 95% confidence interval: 1.34–4.28).

**Conclusions:**

Early sleep intervention should be recommended to infants at 4 months of age as a part of well childcare to improve infant sleep quality.

**Trial Registration:**

Thai Clinical Trials Registry (thaiclinicaltrial.org). Retrospective registered TCTR20230117001 (17/01/2023).

## Introduction

Night waking and a short sleep duration are common issues during the infancy period. According to the normal sleep development, infants aged 3–6 months can consolidate their sleep throughout the night without feeding [1, 2]. A longer duration of nighttime sleep is correlated with older age [3, 4]. Within the first 6 months of life, an infant’s sleep is significantly changing. Infants have the longest sleep period increase rate at 7.2–39 min per month, and have a decreased number of night waking rate at 0.33 wakes per month [5–7]. At 6 months of age, 56% of healthy full-term infants developed sleep regulation, and they could consolidate their sleep up to 8.5 h per night [8]. Nonmaintenance of sleep or a short sleep duration is significantly associated with later sleep issues at 12, 24, and 36 months of age (odd ratio: 6.7, 3.1, and 3.3, respectively) [9] or other issues such as childhood overweight [10], emotional challenges [11], and maternal psychological problems [12–14].

Review studies have shown that good sleep in the early stage of life has several benefits. That is, it can result in a longer night sleep duration and decreased sleep issues, which are associated with positive effects on memory, language, executive function, and cognitive outcomes from toddlerhood to adolescence [15]. Infant sleep intervention reduced the number of night waking, increased daytime and nighttime sleep duration, and promoted independence in going back to sleep after night waking [5, 16–22]. IP Landsem and NB Cheetham [23] evaluated sleep studies on infants aged < 6 months. In particular, 9 of 17 studies focused on infant sleep interventions such as responsive parent intervention, parental cry tolerance, and behavioral–educational sleep intervention. This review showed that interventions focusing on parental education about normal sleep development, bedtime routine consistency, and methods used to develop infant sleep consolidation should be discussed with families within the first 3–6 months of an infant’s life. One important points, the authors suggested that parental education should fit with sociocultural attitude and consistent with routine service, via any types of educational materials.

JA Mindell, A Sadeh, B Wiegand, TH How and DY Goh [24] revealed that race or ethnicity is a factor influencing sleep outcomes. There are only a few studies on early infant sleep intervention among Asians who differ in terms of sleep culture and trajectory. Asian infants had a significantly later bedtime and a shorter total sleep duration than Caucasian infants. Male sex (odds ratio [OR]: 1.5, 95% confidence interval [CI]: 1.3–1.8) and breastfeeding during sleep (OR: 1.3, 95% CI: 1.1–1.5) [25, 26] were associated with a shorter sleeping period or a greater number of night waking.

Even pediatricians and child health personnels are knowledgeable about the importance of promoting good sleep health. However, in real-world settings, several issues should be discussed with the parents within a limited time. As our reviewed, we decided to use a 15-min educational clip to empower parental knowledge about early infant sleep in well-childcare clinic. Further, the efficacy of early sleep intervention before the age of 6 months in Thai infants has been questioned. Hence, the current study aimed to explore the positive effects of a 15-min educational clip about early sleep intervention on sleep outcomes in infants at the age of 6 months.

## Method

### Study design

This was a randomized controlled trial. Eligible participants were randomized into two groups by a computer-generated block of four. One of the authors allocated participants to assign groups and another author provided sleep educational media to intervention group without discussion. The early sleep intervention and usual care groups were appointed at the Vajira hospital well-childcare clinic on different dates. Data were collected by the blinded-research assistant at 4 months of age (before group randomization) and at 6 months via direct or telephone interview. This study followed the criteria of the Consolidated Standards of Reporting Trial (CONSORT) statement for reporting parallel group randomized trial.

### Recruitment of participants

This study was approved by the institutional review board of the faculty of Medicine Vajira hospital (COA 184/64) and retrospectively registered at Thai Clinical Trial Registry (thaiclinicaltrail.org; TCTR20230117001). The time of trial registration was January 16, 2023, research ethical approval was September 26, 2021, and first participant inclusion was November 1, 2021. There was misunderstanding between the first and corresponding authors about trial registration, we immediately registered after recognizing the pitfall. Research teams invited and registered the parents at well-childcare clinic to study. In accordance with the declaration of Helsinki, parents or legal guardians provided a written inform consent. Parents and infants aged 4 months ± 2 weeks who experienced night waking (≥ 3 times/night) were enrolled in this research. Infants with syndrome or genetic diseases, major neurodevelopmental issues (such as brain anomaly, cerebral palsy, and global developmental delay), history of allergy (cow’s milk protein allergy) or colic, a current history of anticonvulsant or sedative use, and a previous history of severe birth asphyxia or low birthweight or birth before 37 weeks of gestational age and twin participants were not included in the analysis. Moreover, parents who had a Center for Epidemiologic Studies-Depression scale (CES-D) score of ≥ 22, those who cannot communicate in Thai language, those whose who did not sleep with an infant, and those who cannot be followed-up were excluded from the study.

### Sample size

Sample size estimates were based on intervention effect on sleep outcome measure, the number of night waking [25] and duration of nighttime sleep [17]. Based on previous study, a sample of 157 participants is required to detect a 30-minute mean difference in sleep duration and 97 participants to detect 20%-reduction of night waking at significant level of 0.05 and 80% power. Considering a 10% possible drop-out, a total of 173 participants per group will be recruited.

### Sleep intervention

A 15-min recorded-media about early sleep intervention was developed by research authors. This media included knowledge about normal sleep development, effects of poor sleep hygiene, safe sleep environment, and methods that can improve infant sleep hygiene.

At baseline (4-month checkup), one of authors provided a 15-min recorded-media to parents of the early sleep intervention group in a group (≤ 10 persons per group) before the well-childcare visit. The of the usual care group had or had not received sleep information at the well-childcare clinic.

### Data assessment

*Sleep quality* was evaluated based on the number of night waking and duration of nighttime sleep. The nighttime period was from 6:01 pm to 6 am and the daytime period from 6:01 am to 6 pm. We collected data on the demographic characteristics of the participants, sleep environment, and infant and parent sleeping data. Information on infant sleep was collected via parental interview at baseline and 2 months after the intervention. A sleep questionnaire designed for this study was used. The content validity of the questionnaire was reviewed and examined by 2 developmental and behavioral pediatrics and 1 pediatric pulmonology. Each item must have the item-objective congruence (IOC) greater than 0.5 before using. The blinded-research assistant was trained to interview about sleep data within the last 7 days, which included sleep–wake time, number of wakening during nighttime, time and duration of each sleep–wake cycles, number of persons per bedroom, bedsharing, and methods used to fall asleep at onset and during nighttime (parental involvement: breastfeeding/bottle-feeding/use of pacifier/holding/padding or touching the baby and self-soothing: thumb sucking/swinging/use of a blanket/no intervention). In intervention group, one of parents who received media, was interviewed. We interviewed the same parents at follow-up visit in both groups.

*Maternal depression* was assessed using CES-D, Thai version [27]. CES-D is a self-reported questionnaire with 20 items. The item scores were as follows: 0, 1, 2, and 3, with a total score of 0–60. A score of ≥ 22 was associated with depression. The reliability Cronbach’s alpha coefficient is 0.86.

### Statistical analysis

Statistical analyses were performed using the Statistical Package for the Social Sciences software version 25 (IBM SPSS Statistics for windows, version 25.0. Armonk, NY: IBM Corp). Continuous variables were expressed as means and standard deviation or median and interquartile range (IQR) if the data had a non-normal distribution. Categorical variables were presented as count and percentages. The baseline characteristics and sleep outcomes of both groups were compared using the chi-square test, independent *t*-test, and Mann–Whitney U test. A *P* value of < 0.05 was considered statistically significant. Variables such as age, sex, breast feeding, family income, parental education, and daytime sleep in the sleep outcome analysis were controlled and presented as adjusted odds ratio (AOR) with 95% confidence interval (95% CI). Incomplete or missing follow-up data were excluded. All analyses were per protocol.

## Results

**Fig. 1:**
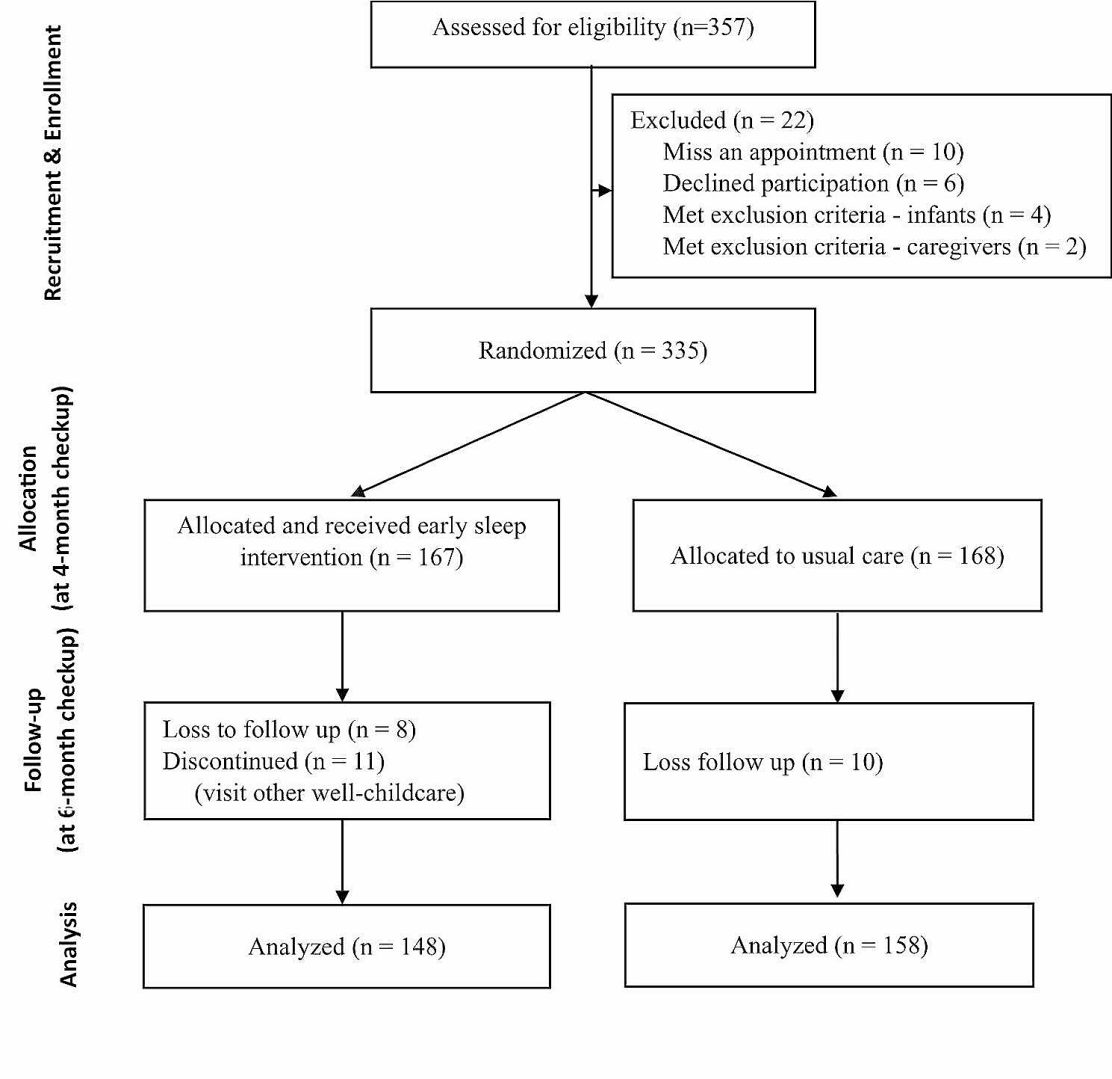
Flowchart of participant selection

In total, 357 infant–mother dyads were recruited, and 22 participants were excluded from the study. Among the remaining 335 participants, 167 were included in the early sleep intervention group and 168 in the usual care group. During the follow-up study, 18 participants were lost to follow-up (*n* = 8 in the early sleep intervention group, *n* = 10 in the usual care group), and 11 participants in the early sleep intervention group refused to be followed-up and visited other well-childcare clinics. Finally, 306 participants were evaluated (*n* = 148 in the early sleep intervention group, *n* = 158 in the usual care group) (Fig. 1).

Table 1 shows the baseline characteristics of infants and parents. Results showed no significant difference in terms of infant sex, nighttime breast feeding, self-soothing ability, mother as the caregiver, caregiver’s age and education, house’s sound environment, and parental depression score. Table 2 presents the baseline characteristics of infant sleep. There was no significant difference in terms of total sleep duration (*P* = .186), total nighttime sleep duration (*P* = .871), and night wake time duration (*P* = .707). The early sleep intervention group had a significantly higher frequency of night waking than the usual care group (*P* < .001).

**Table 1:** Baseline characteristics of infants and parents

Characteristics	Early Sleep Intervention group (*N* = 148)	Usual care group (*N* = 158)	*P* value
**Infants**			
Age (month)	4.04 (0.31)	4.05 (0.26)	0.907
Male	81 (54.7)	85 (53.4)	0.704
Nighttime breast feeding	111 (75.0)	125 (79.1)	0.392
Self-soothing ability	50 (33.8)	45 (28.5)	0.316
Bed sharing	96 (64.9)	100 (63.3)	0.774
**Parents**			
Mother	129 (87.2)	137 (86.7)	0.906
Age (year)	29.97 (7.30)	28.51 (7.16)	0.080
Educational year ≥ 12 years	139 (93.9)	147 (93.0)	0.755
Family income (USD/month)	696.23 (550.02)	612.68 (515.21)	0.511
House environment – quiet	117 (79.1)	123 (77.8)	0.798
Number of persons per bedroom > 3 persons	13 (8.8)	22 (13.9)	0.158
Parental depression score	12.80 (3.25)	12.84 (3.53)	0.936

Data statistics are reported as Mean (SD) or n (%)

Family income is reported as Median (IQR)

**Table 2:** Baseline sleep characteristics of infants and parents

Characteristics	Early Sleep Intervention group (*N* = 148)	Usual care group (*N* = 158)	*P* value
**Infants**			
Total sleep duration			
All-day	810.41 (62.79)	801.27 (57.89)	0.186
Daytime	415.14 (51.67)	405.19 (52.32)	0.096
Nighttime	395.27 (41.95)	396.08 (44.38)	0.871
Night awakening frequency (time/night)	3.10 (0.33)	3.00 (0)	< 0.001
Nighttime wake duration (min/time)	57.71 (32.28)	59.18 (33.10)	0.707
**Parents**			
Nighttime sleep duration (min/time)	193.38 (25.06)	192.15 (24.20)	0.663

Data statistics are reported as Mean (SD)

As shown in Table 3, in terms of the 6-month outcome, the early sleep intervention group had a longer duration of total nighttime sleep (585.20 ± 80.38 vs. 496.14 ± 87.78 min, *P* < .001) and night sleep period (213.11 ± 64.54 vs. 170.20 ± 56.98 min, *P* < .001), shorter duration of night waking time (61.01 ± 36.38 vs. 89.72 ± 45.54 min, *P* < .001), and lower number of night waking (1.93 ± 0.69 vs. 2.10 ± 0.67, *P* = .025) than the usual care group. After adjusting for covariates, the early sleep intervention group had a longer nighttime sleep duration (sleep ≥ 4 h/time) than the usual care group (2.39 times; AOR 2.39, 95% CI: 1.34–4.28) (Table 4).

**Table 3:** Outcomes of infant nighttime sleep quality assessed at 6-month age

Outcomes	Early Sleep Intervention group (*N* = 148)	Usual care group (*N* = 158)	*P* value
Total nighttime sleep duration (min)	585.20 (80.38)	496.14 (87.78)	< 0.001
Sleep duration per time(min)	213.11 (64.54)	170.20 (56.98)	< 0.001
Night awakening frequency (time/night)	1.93 (0.69)	2.10 (0.67)	0.025
Night wake time duration (min/time)	61.01 (36.38)	89.72 (45.54)	< 0.001
Night awakening ≤ 2 times	120 (81.1)	114 (72.2)	0.006
Sleep duration ≥ 4 h/time	42 (28.4)	22 (13.9)	0.002

Data statistics are reported as Mean (SD) or n (%)

**Table 4:** Logistic regression analysis of infant sleep quality

Characteristics	Early sleep intervention group VS. Usual care group (reference)		
	AOR^a^	95% CI	*P* value
Night awakening ≤ 2 times	1.62	0.92–2.84	0.095
Nighttime sleep duration ≥ 4 h/time	2.39	1.34–4.28	0.003

^a^Adjusted odds ratio (AOR) with age, sex, breast feeding, family income, parental education and daytime sleep hours

## Discussion

### Infant sleep quality

This study found that early sleep intervention at the age of 4 months significantly improved sleep quality at the age of 6 months in infants. Providing a 15-min sleep education media to parents resulted in a longer total nighttime sleep duration (approximately 89 min) and a lower number of night waking in the intervention group compared with the usual care group. The early sleep intervention group had a higher proportion of infants who had prolonged night sleep (> 4 h per time) than the usual care group (28.4% vs. 13.9%). The effect of sleep intervention significantly differed after adjusting for covariate factors (AOR: 2.39, 95% CI: 1.34–4.28). Our results supported the study of IP Landsem and NB Cheetham [23], which showed the importance of providing encouragement to parents with infants who experienced sleep issues before the age of 6 months.

### Comparison with previous studies

The nighttime sleep duration of infants in this study was similar to that in the randomized controlled study by IM Paul, JS Savage, S Anzman-Frasca, ME Marini, JA Mindell and LL Birch [17]. That is, the early sleep intervention group had a longer total nighttime sleep duration than the control group. However, other studies (BC Galland, RM Sayers, SL Cameron, AR Gray, AM Heath, JA Lawrence, A Newlands, BJ Taylor and RW Taylor [28], IS Santos, B Del-Ponte, L Tovo-Rodrigues, CS Halal, A Matijasevich, S Cruz, L Anselmi, MF Silveira, PRC Hallal and DG Bassani [29]) found that early sleep intervention did not significantly enhance sleep outcomes between two groups based on maternal reports. The early sleep intervention and usually care groups in this study had a higher percentage of breastfeeding during nighttime (75.4–79.2%) than those in previous studies on Thai/Asian infants (34.9–56.9%) [25, 26]. Breastfeeding was associated with a greater number of night waking. This finding was in contrast to that of previously published studies [17, 28, 29], which showed that early sleep intervention had no significant effect on the number of night waking. Moreover, the current study found that the early sleep intervention group had a significantly lower night waking frequency (1.93 ± 0.69 vs. 2.10 ± 0.67, *P* = .025) than the usual care group. Based on previous randomized controlled studies, 4–6% of the Asian populations may have ethnic or sleep cultural differences.

The early sleep intervention and usual care groups had a low proportion of 6-month-old infants who slept > 4 h/period (28.4 vs. 13.9). Further, only one infant from the early sleep intervention group slept for > 6 h/period (data not shown). The development of sleep consolidation in most infants was slower than expected. This result may be associated with parental sleep duration, late onset of parental sleep, or family socioeconomic status. Hence, this notion should be further explored.

### Limitations

Our intervention was a simplified method and could be provided in all well-child clinics. We used a 15-min educational video clip regarding the benefits of good sleep, normal infant sleeping duration, and methods that can be used to increase nighttime sleeping hours and facilitate a safe sleep to educate parents of 4-month-old infants. One research assistant who was blinded to the participant’s group was trained to perform interviews. Each group was appointed separately to decrease contamination.

The current study had some limitations. Similar studies on infant sleep, number of night waking, and sleep duration were based on parental report. The current analysis method is feasible. However, recall bias might have existed, and inaccurate information on sleep outcomes could have been obtained with this method compared with the gold-standard method. M Camerota, KP Tully, M Grimes, N Gueron-Sela and CB Propper [30] suggested sleep questionnaire using beware underestimated 1.38 time of night waking and overestimated 32 min of infant sleep duration when compared with videosomnography. However, according to subjective parental reports, infant sleep duration and number of night waking were correlated with actigraphy results particularly in breast-fed infants [31, 32]. One of limitations was natural exposure of sleep knowledge from social media or others, we did not explore or control about this. This may enhance the effect of intervention. The long-term efficacy of intervention is another limitation. Finally, considering statistics, we excluded missing outcome data from analysis, even though the result showed no difference in baseline characteristics, but interpretation of final outcomes should be aware of type I error.

## Conclusions

Early sleep intervention had positive effects. The intervention focused on parental education about normal infant sleeping patterns and the promotion of healthy sleep. Further, information on early sleep intervention should be provided to families before the age of 6 months of infants. Nevertheless, further studies must be performed to evaluate self-soothing abilities or abilities to develop any behaviors that infant use to regulate themselves to sleep without parental involvement after early sleep intervention or the long-term outcomes of the intervention in Asian populations.

## Data Availability

The datasets used and/or analysed during the current study available from corresponding author on reasonable request.
